# Supplementary material for: HIVprotI: an integrated web based platform for prediction and design of HIV proteins inhibitors
Source: J Cheminform. 2018 Mar 9;10:12. doi: 10.1186/s13321-018-0266-y (PMC5845081; doi:10.1186/s13321-018-0266-y)
Supplement: Supplementary file 2 — Additional file 2. Source code of HIVProtI web server. [file 13321_2018_266_MOESM2_ESM.zip › HIVprotI_Source-code/hivprotI/weka-3-6-6/doc/weka/clusterers/forOPTICSAndDBScan/OPTICS_GUI/GraphPanel.html]

GraphPanel


---


|  |  |  |
| --- | --- | --- |
| **Overview** \| **Package** \| **Class** \| **Tree** \| **Deprecated** \| **Index** \| **Help** \| **Weka's home** | |  |
| PREV CLASS   **NEXT CLASS** | **FRAMES**    **NO FRAMES**     **All Classes** |  |
| SUMMARY: NESTED \| FIELD \| CONSTR \| METHOD | DETAIL: FIELD \| CONSTR \| METHOD |  |


---


## weka.clusterers.forOPTICSAndDBScan.OPTICS\_GUI Class GraphPanel

```
java.lang.Object
  java.awt.Component
      java.awt.Container
          javax.swing.JComponent
              weka.clusterers.forOPTICSAndDBScan.OPTICS_GUI.GraphPanel
```

**All Implemented Interfaces:**: java.awt.image.ImageObserver, java.awt.MenuContainer, java.io.Serializable, RevisionHandler

---

``` public class GraphPanel extends javax.swing.JComponent implements RevisionHandler ```

GraphPanel.java

**Version:**
:   $Revision: 1.4 $

**Author:**
:   Zhanna Melnikova-Albrecht (melnikov@cip.ifi.lmu.de), Rainer Holzmann (holzmann@cip.ifi.lmu.de)

**See Also:**: Serialized Form

---

**Nested Class Summary**

**Nested classes/interfaces inherited from class javax.swing.JComponent**

`javax.swing.JComponent.AccessibleJComponent`

**Nested classes/interfaces inherited from class java.awt.Component**

`java.awt.Component.BaselineResizeBehavior`


**Field Summary**

**Fields inherited from class javax.swing.JComponent**

`TOOL_TIP_TEXT_KEY, UNDEFINED_CONDITION, WHEN_ANCESTOR_OF_FOCUSED_COMPONENT, WHEN_FOCUSED, WHEN_IN_FOCUSED_WINDOW`

**Fields inherited from class java.awt.Component**

`BOTTOM_ALIGNMENT, CENTER_ALIGNMENT, LEFT_ALIGNMENT, RIGHT_ALIGNMENT, TOP_ALIGNMENT`

**Fields inherited from interface java.awt.image.ImageObserver**

`ABORT, ALLBITS, ERROR, FRAMEBITS, HEIGHT, PROPERTIES, SOMEBITS, WIDTH`


**Constructor Summary**

`GraphPanel(FastVector resultVector,
int verticalAdjustment,
boolean showCoreDistances,
boolean showReachbilityDistances)`


| **Method Summary** | |
| --- | --- |
| `void` | `adjustSize(SERObject serObject)`             Adjusts the size of this panel in respect of the shown content |
| `java.lang.String` | `getRevision()`             Returns the revision string. |
| `boolean` | `isShowCoreDistances()`             Returns the flag for showCoreDistances |
| `boolean` | `isShowReachabilityDistances()`             Returns the flag for showReachabilityDistances |
| `void` | `setCoreDistanceColor(java.awt.Color coreDistanceColor)`             Sets a new color for the coreDistance |
| `void` | `setNewToolTip(java.lang.String toolTip)`             Displays a toolTip for the selected DataObject |
| `void` | `setReachabilityDistanceColor(java.awt.Color reachabilityDistanceColor)`             Sets a new color for the reachabilityDistance |
| `void` | `setResultVector(FastVector resultVector)`             Sets a new resultVector |
| `void` | `setShowCoreDistances(boolean showCoreDistances)`             Sets the flag for showCoreDistances |
| `void` | `setShowReachabilityDistances(boolean showReachabilityDistances)`             Sets the flag for showReachabilityDistances |
| `void` | `setVerticalAdjustment(int verticalAdjustment)`             Sets a new value for the vertical verticalAdjustment |

**Methods inherited from class javax.swing.JComponent**

`addAncestorListener, addNotify, addVetoableChangeListener, computeVisibleRect, contains, createToolTip, disable, enable, firePropertyChange, firePropertyChange, firePropertyChange, getAccessibleContext, getActionForKeyStroke, getActionMap, getAlignmentX, getAlignmentY, getAncestorListeners, getAutoscrolls, getBaseline, getBaselineResizeBehavior, getBorder, getBounds, getClientProperty, getComponentPopupMenu, getConditionForKeyStroke, getDebugGraphicsOptions, getDefaultLocale, getFontMetrics, getGraphics, getHeight, getInheritsPopupMenu, getInputMap, getInputMap, getInputVerifier, getInsets, getInsets, getListeners, getLocation, getMaximumSize, getMinimumSize, getNextFocusableComponent, getPopupLocation, getPreferredSize, getRegisteredKeyStrokes, getRootPane, getSize, getToolTipLocation, getToolTipText, getToolTipText, getTopLevelAncestor, getTransferHandler, getUIClassID, getVerifyInputWhenFocusTarget, getVetoableChangeListeners, getVisibleRect, getWidth, getX, getY, grabFocus, isDoubleBuffered, isLightweightComponent, isManagingFocus, isOpaque, isOptimizedDrawingEnabled, isPaintingForPrint, isPaintingTile, isRequestFocusEnabled, isValidateRoot, paint, paintImmediately, paintImmediately, print, printAll, putClientProperty, registerKeyboardAction, registerKeyboardAction, removeAncestorListener, removeNotify, removeVetoableChangeListener, repaint, repaint, requestDefaultFocus, requestFocus, requestFocus, requestFocusInWindow, resetKeyboardActions, reshape, revalidate, scrollRectToVisible, setActionMap, setAlignmentX, setAlignmentY, setAutoscrolls, setBackground, setBorder, setComponentPopupMenu, setDebugGraphicsOptions, setDefaultLocale, setDoubleBuffered, setEnabled, setFocusTraversalKeys, setFont, setForeground, setInheritsPopupMenu, setInputMap, setInputVerifier, setMaximumSize, setMinimumSize, setNextFocusableComponent, setOpaque, setPreferredSize, setRequestFocusEnabled, setToolTipText, setTransferHandler, setVerifyInputWhenFocusTarget, setVisible, unregisterKeyboardAction, update, updateUI`

**Methods inherited from class java.awt.Container**

`add, add, add, add, add, addContainerListener, addPropertyChangeListener, addPropertyChangeListener, applyComponentOrientation, areFocusTraversalKeysSet, countComponents, deliverEvent, doLayout, findComponentAt, findComponentAt, getComponent, getComponentAt, getComponentAt, getComponentCount, getComponents, getComponentZOrder, getContainerListeners, getFocusTraversalKeys, getFocusTraversalPolicy, getLayout, getMousePosition, insets, invalidate, isAncestorOf, isFocusCycleRoot, isFocusCycleRoot, isFocusTraversalPolicyProvider, isFocusTraversalPolicySet, layout, list, list, locate, minimumSize, paintComponents, preferredSize, printComponents, remove, remove, removeAll, removeContainerListener, setComponentZOrder, setFocusCycleRoot, setFocusTraversalPolicy, setFocusTraversalPolicyProvider, setLayout, transferFocusBackward, transferFocusDownCycle, validate`

**Methods inherited from class java.awt.Component**

`action, add, addComponentListener, addFocusListener, addHierarchyBoundsListener, addHierarchyListener, addInputMethodListener, addKeyListener, addMouseListener, addMouseMotionListener, addMouseWheelListener, bounds, checkImage, checkImage, contains, createImage, createImage, createVolatileImage, createVolatileImage, dispatchEvent, enable, enableInputMethods, firePropertyChange, firePropertyChange, firePropertyChange, firePropertyChange, firePropertyChange, getBackground, getBounds, getColorModel, getComponentListeners, getComponentOrientation, getCursor, getDropTarget, getFocusCycleRootAncestor, getFocusListeners, getFocusTraversalKeysEnabled, getFont, getForeground, getGraphicsConfiguration, getHierarchyBoundsListeners, getHierarchyListeners, getIgnoreRepaint, getInputContext, getInputMethodListeners, getInputMethodRequests, getKeyListeners, getLocale, getLocation, getLocationOnScreen, getMouseListeners, getMouseMotionListeners, getMousePosition, getMouseWheelListeners, getName, getParent, getPeer, getPropertyChangeListeners, getPropertyChangeListeners, getSize, getToolkit, getTreeLock, gotFocus, handleEvent, hasFocus, hide, imageUpdate, inside, isBackgroundSet, isCursorSet, isDisplayable, isEnabled, isFocusable, isFocusOwner, isFocusTraversable, isFontSet, isForegroundSet, isLightweight, isMaximumSizeSet, isMinimumSizeSet, isPreferredSizeSet, isShowing, isValid, isVisible, keyDown, keyUp, list, list, list, location, lostFocus, mouseDown, mouseDrag, mouseEnter, mouseExit, mouseMove, mouseUp, move, nextFocus, paintAll, postEvent, prepareImage, prepareImage, remove, removeComponentListener, removeFocusListener, removeHierarchyBoundsListener, removeHierarchyListener, removeInputMethodListener, removeKeyListener, removeMouseListener, removeMouseMotionListener, removeMouseWheelListener, removePropertyChangeListener, removePropertyChangeListener, repaint, repaint, repaint, resize, resize, setBounds, setBounds, setComponentOrientation, setCursor, setDropTarget, setFocusable, setFocusTraversalKeysEnabled, setIgnoreRepaint, setLocale, setLocation, setLocation, setName, setSize, setSize, show, show, size, toString, transferFocus, transferFocusUpCycle`

**Methods inherited from class java.lang.Object**

`equals, getClass, hashCode, notify, notifyAll, wait, wait, wait`

**Constructor Detail**

### GraphPanel

```
public GraphPanel(FastVector resultVector,
                  int verticalAdjustment,
                  boolean showCoreDistances,
                  boolean showReachbilityDistances)
```


**Method Detail**

### setResultVector

```
public void setResultVector(FastVector resultVector)
```

:   Sets a new resultVector

    :   **Parameters:**: `resultVector` -

---


### setNewToolTip

```
public void setNewToolTip(java.lang.String toolTip)
```

:   Displays a toolTip for the selected DataObject

    :   **Parameters:**: `toolTip` -

---


### adjustSize

```
public void adjustSize(SERObject serObject)
```

:   Adjusts the size of this panel in respect of the shown content

    :   **Parameters:**: `serObject` - SERObject that contains the OPTICS clustering results

---


### isShowCoreDistances

```
public boolean isShowCoreDistances()
```

:   Returns the flag for showCoreDistances

    :   **Returns:**: True or false

---


### setShowCoreDistances

```
public void setShowCoreDistances(boolean showCoreDistances)
```

:   Sets the flag for showCoreDistances

    :   **Parameters:**: `showCoreDistances` -

---


### isShowReachabilityDistances

```
public boolean isShowReachabilityDistances()
```

:   Returns the flag for showReachabilityDistances

    :   **Returns:**: True or false

---


### setShowReachabilityDistances

```
public void setShowReachabilityDistances(boolean showReachabilityDistances)
```

:   Sets the flag for showReachabilityDistances

    :   **Parameters:**: `showReachabilityDistances` -

---


### setVerticalAdjustment

```
public void setVerticalAdjustment(int verticalAdjustment)
```

:   Sets a new value for the vertical verticalAdjustment

    :   **Parameters:**: `verticalAdjustment` -

---


### setCoreDistanceColor

```
public void setCoreDistanceColor(java.awt.Color coreDistanceColor)
```

:   Sets a new color for the coreDistance

    :   **Parameters:**: `coreDistanceColor` -

---


### setReachabilityDistanceColor

```
public void setReachabilityDistanceColor(java.awt.Color reachabilityDistanceColor)
```

:   Sets a new color for the reachabilityDistance

    :   **Parameters:**: `reachabilityDistanceColor` -

---


### getRevision

```
public java.lang.String getRevision()
```

:   Returns the revision string.

    :   **Specified by:**: `getRevision` in interface `RevisionHandler`
    :   **Returns:**: the revision


---


|  |  |  |
| --- | --- | --- |
| **Overview** \| **Package** \| **Class** \| **Tree** \| **Deprecated** \| **Index** \| **Help** \| **Weka's home** | |  |
| PREV CLASS   **NEXT CLASS** | **FRAMES**    **NO FRAMES**     **All Classes** |  |
| SUMMARY: NESTED \| FIELD \| CONSTR \| METHOD | DETAIL: FIELD \| CONSTR \| METHOD |  |


---
